# Numbers of Granule Cells and GABAergic Boutons Are Correlated in Shrunken Sclerotic Hippocampi of Sea Lions with Temporal Lobe Epilepsy

**DOI:** 10.1523/ENEURO.0389-25.2026

**Published:** 2026-03-24

**Authors:** Megan Wyeth, David D. R. Krucik, Chloé J. Thorbrogger, Cara Field, Paul S. Buckmaster

**Affiliations:** ^1^Department of Comparative Medicine, Stanford University, Palo Alto, California 94304; ^2^The Marine Mammal Center, Sausalito, California 94965; ^3^Department of Neurology & Neurological Sciences, Stanford University, Palo Alto, California 94304

**Keywords:** dentate gyrus, GABAergic, granule cell, interneuron, stereology, terminal

## Abstract

A possible mechanism of temporal lobe epilepsy is insufficient inhibition of hippocampal dentate granule cells. Precipitating injuries that kill interneurons in the dentate gyrus might result in fewer inhibitory synapses with granule cells. To test this hypothesis, previous studies evaluated numbers or densities of interneurons, γ-amino butyric acid (GABA)ergic boutons, and inhibitory synapses in tissue from human patients with temporal lobe epilepsy and rodent models. However, those studies have limitations. Some of those limitations can be addressed by a large animal model. Sea lions (*Zalophus californianus*) can develop temporal lobe epilepsy naturally. Like humans, epileptic sea lions exhibit bilateral or unilateral hippocampal sclerosis (neuron loss) with granule cell vulnerability, but sea lions permit optimal tissue preservation and sampling, and good control subjects. To label interneuron cell bodies and GABAergic synaptic boutons, sea lion hippocampal tissue from both sexes was processed with immunohistochemistry for glutamic acid decarboxylase (GAD) and vesicular GABA transporter. Stereological techniques were used to evaluate the dentate gyrus of the entire hippocampus. Numbers of granule cells, GAD cells, and GABAergic boutons were substantially reduced in shrunken, sclerotic hippocampi. However, numbers of GABAergic boutons and granule cells were correlated. These findings indicate that, despite losses, numbers of GABAergic boutons scale with numbers of surviving granule cells.

## Significance Statement

Temporal lobe epilepsy is a challenging clinical problem. Electrophysiological studies reveal that hippocampal dentate granule cells are insufficiently inhibited and hyperexcitable in epileptic tissue from humans and rodent models. The present stereological analysis of a large animal model (sea lions) found no evidence for disproportionate loss of dentate gyrus GABAergic boutons in temporal lobe epilepsy. These data suggest reduced inhibition of granule cells is attributable to something other than too few GABAergic boutons.

## Introduction

One of 26 people in the United States develops epilepsy at some time in their life (Hesdorffer et al., 2011). Temporal lobe epilepsy is one of the most common types (Engel et al., 1997). The hippocampus is the most frequent site of seizure initiation (Quesney, 1986; Spencer et al., 1987, 1990; Sperling and O’Connor, 1989; Duckrow and Spencer, 1992; Spencer and Spencer, 1994; Masukawa et al., 1995; King et al., 1997; Wennberg et al., 2002). Normally, the hippocampal dentate gyrus has gate-like properties that resist seizure activity (Lothman et al., 1991; Heinemann et al., 1992; Krook-Magnuson et al., 2015). However, in patients with temporal lobe epilepsy, the dentate gyrus displays neuropathology (Margerison and Corsellis, 1966), including loss of inhibitory interneurons (de Lanerolle et al., 1989; Sloviter et al., 1991; Mathern et al., 1995; Zhu et al., 1997; Maglóczky et al., 2000; Andrioli et al., 2007; Tóth et al., 2010).

In hippocampal slices prepared from tissue resected to treat patients, the dentate gyrus can generate seizure activity (Gabriel et al., 2004; Jandová et al., 2006), and granule cells are hyperexcitable (Isokawa et al., 1991; Isokawa and Levesque, 1991) largely because of reduced inhibition (Masukawa et al., 1989; Franck et al., 1995; Isokawa, 1996; Williamson et al., 1999). Deficits of granule cell inhibition also have been reported in rodent models of temporal lobe epilepsy (Sayin et al., 2003; Pathak et al., 2007; Sun et al., 2014; Dengler et al., 2017). For example, γ-amino butyric acid (GABA)ergic synaptic input to granule cells, measured as miniature inhibitory postsynaptic current frequency, is markedly reduced (Kobayashi and Buckmaster, 2003; Shao and Dudek, 2005; Sun et al., 2007).

Interneuron loss may reduce the number of GABAergic synapses with granule cells, resulting in less inhibition, hyperexcitability, and seizures. The hypothesis is consistent with efficacy of antiseizure drugs that augment GABAergic synaptic function (Greenfield, 2013), with evidence that promoting inhibitory synaptogenesis decreases epileptiform activity (Adel et al., 2023), and with reduced seizure frequency after transplanting GABAergic cells into the hippocampus in rodent models of temporal lobe epilepsy (Hunt et al., 2013; Casalia et al., 2017; Upadhya et al., 2019; Zhu et al., 2023). Contrary to the hypothesis, studies of tissue from patients with temporal lobe epilepsy (Babb et al., 1989; Wittner et al., 2001; Wittner and Maglóczky, 2017; Alhourani et al., 2020) and rodent models (Thind et al., 2010) have reported more, not fewer, GABAergic boutons or inhibitory synapses per granule cell. However, the evidence against the hypothesis has limitations. Some of those limitations can be addressed by a large animal model.

California sea lions are a large animal model of temporal lobe epilepsy (Buckmaster, 2017). Adult sea lions (*Zalophus californianus*) weigh up to 220 (females) or 850 pounds (males). Their gyrencephalic brain is one-quarter the size of a human brain. During naturally occurring oceanic algal blooms, sea lions are exposed by diet to the kainic acid receptor agonist domoic acid, which can result in status epilepticus. Some surviving sea lions develop temporal lobe epilepsy. Epileptic sea lions have a poor prognosis and are euthanized to avoid suffering. These sea lions reproduce the neuropathology of human patients, including unilateral hippocampal sclerosis in many cases and partial loss of granule cells (Buckmaster et al., 2014; Cameron et al., 2019), which is an advantage over rodent models. Sea lion tissue is not limited by constraints of human studies including small sample sizes, inadequate controls, limited tissue sampling, or compromised tissue preservation. The present study used immunohistochemistry and stereological techniques to evaluate GABAergic neurons and boutons in the dentate gyrus of entire hippocampi in sea lions with temporal lobe epilepsy to test the hypothesis that there is disproportionate loss of GABAergic boutons relative to granule cells.

## Materials and Methods

This experiment used brain tissue of sea lions (*Z. californianus*) from a previous study that reports details on subjects and methods of perfusion and sectioning (Cameron et al., 2019). Subjects included those stranded along the California coast who were admitted to The Marine Mammal Center for rehabilitation but did not respond to treatment and were euthanized due to poor prognosis for release. Of 29 sea lions in the present study, 19 were female (sex was undetermined in one). Ages included 1 pup, 2 yearlings, 4 juveniles, 6 subadults, and 16 adults. Hippocampal sclerosis was identified by severe hilar neuron loss (Buckmaster et al., 2014; Cameron et al., 2019). Spontaneous seizures were witnessed in 9 of 16 sea lions with hippocampal sclerosis. Control sea lions (*n* = 13) did not have hippocampal sclerosis, and reasons for euthanasia included trauma and infection. Immediately upon euthanasia by pentobarbital overdose, sea lions were perfused through the ascending aorta (1 L/min) with 0.9% NaCl for 2 min, followed by 4% formaldehyde in phosphate buffer (PB) for 30 min. Brains were bisected and each hemisphere cut into ∼2-cm-thick coronal blocks for postfixing in 4% formaldehyde with 30% sucrose in PB at 4°C. After equilibrating for a week, blocks were frozen in isopentane and stored at −80°C. Sections (40 µm) from a sliding microtome were stored in cryoprotective solution at −20°C until processing for immunohistochemistry.

**Figure 1.**
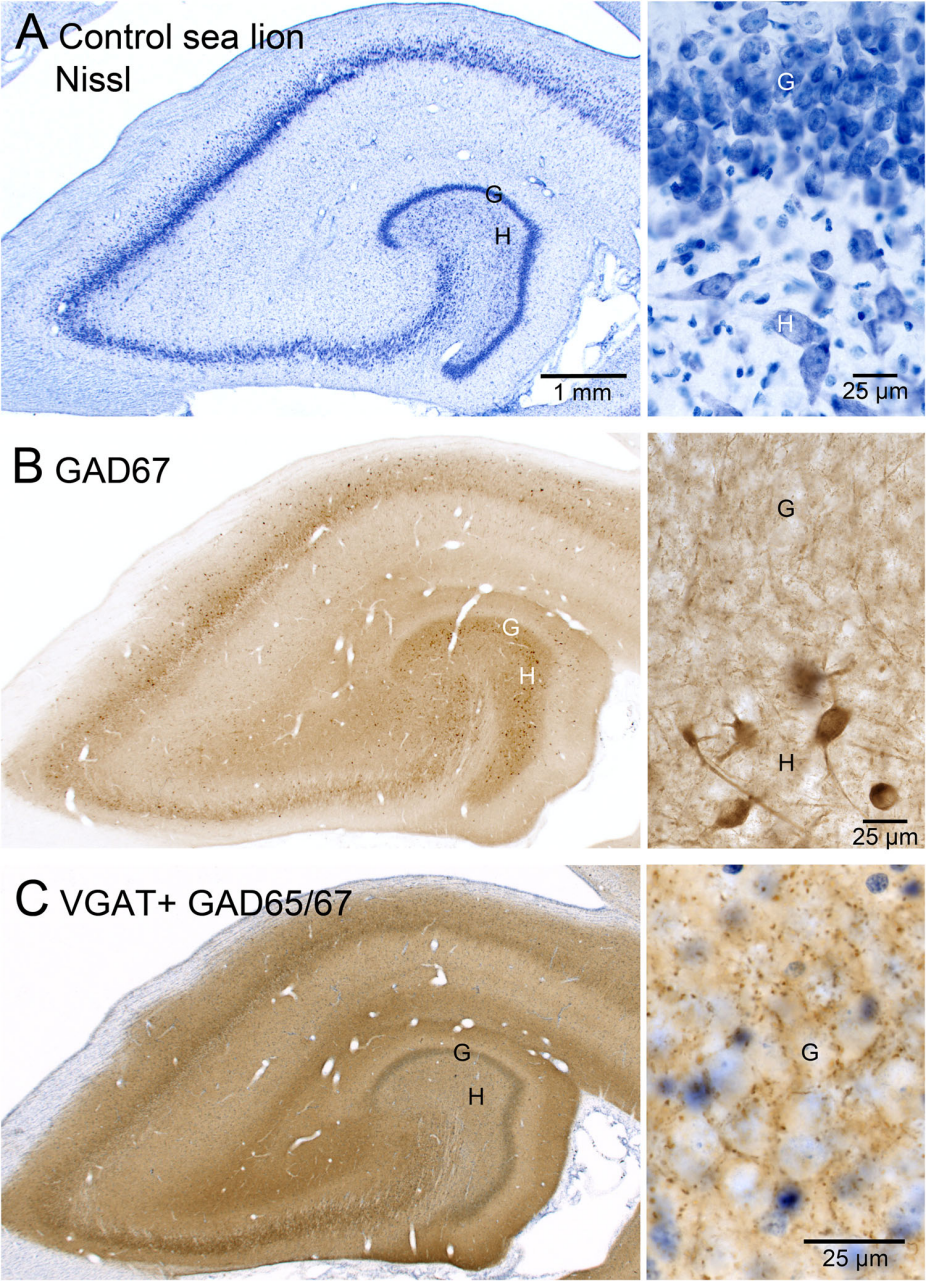
Adult female control sea lion hippocampus. Nissl stain (***A***), glutamic acid decarboxylase (GAD)67-immunoreactivity to label cell bodies (***B***), and vesicular γ-amino butyric acid vesicular transporter (VGAT) plus GAD65/67-immunoreactivity to label synaptic boutons (***C***).

Both hippocampi of all subjects were evaluated with three stains: the Nissl stain thionin, glutamic acid decarboxylase (GAD)-immunoreactivity to visualize interneuron cell bodies, and GAD- and vesicular GABA transporter (VGAT)-immunoreactivity to identify GABAergic synaptic boutons (Fig. 1). Sections processed for bouton staining were lightly counterstained with thionin to facilitate granule cell layer identification.

Previously, a 1-in-40 series of coronal sections (starting at a random point near the temporal pole, on average 10.3 sections/hippocampus) was stained with thionin to stereologically estimate the number of granule cells per hippocampus and identify sclerotic hippocampi based on severe hilar neuron loss (Cameron et al., 2019; Table 1). Those data were used in the present study, which also processed another 1-in-40 series of sections for GAD67-immunoreactivity using a staining protocol optimized to label interneuron cell bodies. Free-floating sections were washed in 1% hydrogen peroxide in 0.05 M Tris-buffered saline (TBS), rinsed, and preincubated in blocking solution containing 3% normal goat serum (NGS) and 2% bovine serum albumin (BSA) in TBS. Sections were transferred to a primary solution with anti-GAD67 (1:1,000, Millipore No. MAB5406), 1% NGS, and 0.2% BSA for 1 week at 4°C. Sections were rinsed and incubated for 2 h in a secondary solution composed of goat anti-mouse (1:500, Vector Laboratories No. BP-9200, RRID:AB_2827937) and 2% BSA in TBS, followed by 2 h in Vectastain Elite ABC-HRP (1:500 in TBS, Vector Laboratories) with 2% BSA. Finally, sections were processed for visualization with 2% diaminobenzidine tetrahydrochloride in 0.1 M Tris buffer, mounted, dehydrated, and coverslipped.

**Table 1.** Parameters of optical fractionator analyses

	Counting frame (µm)	Counting grid (µm)	Dissector height	Average sections analyzed	Average caps counted	Coefficient of variation	Mean coefficient of error^a^
Granule cells^b^	10 × 10	150 × 150	Total section thickness	10	152	0.72	0.14
GAD cells	100 × 100	500 × 500 or 350 × 350^c^	Total section thickness	10	198	0.54	0.07
GABAergic boutons—sea lions	5 × 5	800 × 800	1 µm	10	1,079	0.59	0.07
GABAergic boutons—rats	5 × 5	400 × 400	1 µm	10	921	0.10	0.05

a Calculated according to West et al. (1991).

b From Cameron et al. (2019).

c Shrunken hippocampi were analyzed with a counting grid of 350 × 350 µm so enough GAD cells could be sampled.

Another 1-in-40 series of sections was processed with a staining protocol optimized to label GABAergic synaptic boutons. In the interest of labeling GABAergic boutons as completely as possible, pilot studies tested numerous antibodies in sea lion tissue. Antibodies that produced optimal labeling of boutons were to GAD65/67 (1:3,000, L127/12, UC Davis/NIH NeuroMab Facility) and to VGAT (1:2000, MSFR106160, Frontier Institute). Previous studies revealed robust GABAergic bouton labeling when antibodies to GAD and VGAT were combined (Alhourani et al., 2020). In the present study, tissue was processed with both antibodies combined to avoid missing boutons that might be labeled only by one. The immunohistochemistry protocol was the same as for GAD67, apart from the addition of 0.3% triton to the blocking, primary, secondary, and ABC solutions, and the inclusion of goat anti-guinea pig serum (1:500, Vector Laboratories No. BA-7000, RRID:AB_2336132) in the secondary solution.

### Antibody characterization

The well-established monoclonal antibody to GAD67 used here (clone 1G10.2, RRID:AB_2278725) was raised in mouse against the 67 kDa human isoform (amino acids 4-101). It produces a single band by immunoblot and has no cross-reactivity with the 65 kDa isoform from rat brain (Fong et al., 2005). Immunoreactivity was eliminated in the brain of a Gad1 knock-out rat (Fujihara et al., 2020). Cell labeling was similar to adjacent sections of rat brainstem processed for in situ hybridization (Fong et al., 2005). Importantly, the labeling in sea lion produced the expected pattern of cell bodies in the dentate gyrus based on in situ hybridization in rat (Houser and Esclapez, 1994; Esclapez and Houser, 1999).

Polyclonal anti-VGAT (RRID:AB_2571624) was raised in guinea pig against a fusion protein for mouse VGAT (amino acids 31-112). Immunoblot detected a single protein band at ∼57 kDa (Fukudome et al., 2004) as expected (Chaudhry et al., 1998). The expression in interneuron boutons of sea lion hippocampus corresponded to the labeling pattern in rats (Chaudhry et al., 1998; Boulland et al., 2007).

Monoclonal anti-GAD65/67 (clone L127/12, RRID:AB_2756510) was raised in mouse against the full-length (amino acids 1-594) fusion protein of human GAD67. It has a molecular weight of ∼70 kDa and cross-reacts with GAD65 (60% identity, highest in the C-terminus, manufacturer's datasheet). The staining pattern of interneuron boutons in sea lion hippocampus matched previous reports in rats (Ribak et al., 1978; Fukuda et al., 1998).

### Microscopic analysis

To analyze the distribution of interneurons across layers and along the septotemporal axis of the dentate gyrus, all GAD-positive cell body profiles were marked using Stereo Investigator (MBF Bioscience). To estimate the number of dentate gyrus GAD cells, the optical fractionator method was used (Table 1). Contours were drawn around the entire dentate gyrus: hilus, granule cell layer, and molecular layer. Cells were visualized with a 63× Plan-Apochromat 1.4 na lens (Zeiss). Cell bodies that were not cut at the section surface and began coming into focus while focusing down through the section were counted. Counting grid size depended on hippocampus size. Shrunken, sclerotic hippocampi were evaluated with a smaller grid so that enough cells could be counted. The Cavalieri method was used with area measurements from contours drawn around the dentate gyrus to estimate dentate gyrus volume.

**Figure 2.**
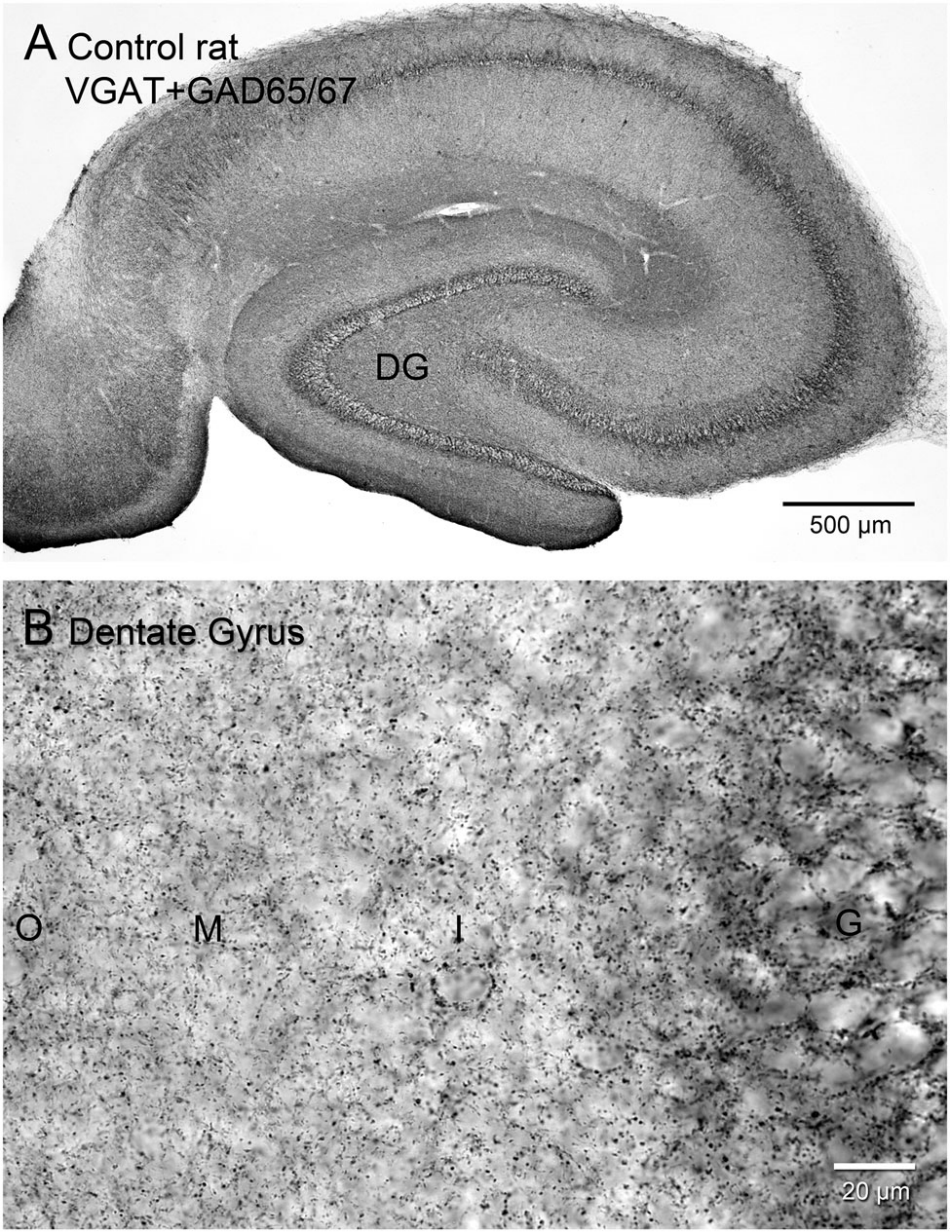
Control rat hippocampus processed for vesicular γ-amino butyric acid transporter (VGAT) plus glutamic acid decarboxylase (GAD)65/67-immunoreactivity to label synaptic boutons at low (***A***) and high magnification (***B***). DG, dentate gyrus; G, granule cell layer; I, inner molecular layer; M, middle molecular layer; O, outer molecular layer.

For GABAergic boutons, an optical fractionator protocol was developed and validated using rat hippocampi to ensure accuracy (Table 1). For rats the number of stereologically estimated immunolabeled gephyrin punctae and electron microscopically identified GABA-immunopositive synapses have been reported (Thind et al., 2010). Experiments were approved by the Stanford University Institutional Animal Care and Use Committee and conducted in compliance with the US National Research Council's “Guide for the Care and Use of Laboratory Animals” and the US Public Health Service's “Policy on Humane Care and Use of Laboratory Animals” and “Guide for the Care and Use of Laboratory Animals.” In the present study, Sprague Dawley rats, 2 females, 2 males, 7 months old, were euthanized with pentobarbital (>100 mg/kg, i.p.) and perfused through the ascending aorta (30 ml/min) for 1 min with 0.9% NaCl and 30 min with 4% formaldehyde in PB. At 4°C, brains were postfixed overnight and then equilibrated in 30% sucrose in PB. Hippocampi were dissected from the brain, straightened, frozen, and sectioned from the septal pole to the temporal pole with a sliding microtome set at 40 μm. Starting at a random point near the septal pole, a 1-in-24 series of sections was sampled and processed with a staining protocol optimized to label GABAergic synaptic boutons in rat tissue (Fig. 2), using the same antibodies as for sea lions: one to GAD65/67 (1:60,000, L127/12, UC Davis/NIH NeuroMab Facility) and the other to VGAT (1:3,000, MSFR106160, Frontier Institute), but preceded by antigen retrieval in 0.5 M citrate buffer with 1% NaCl (pH 8.6) at 90°C for 70 min and avidin/biotin blocking. Additionally, triton was omitted necessitating room temperature incubations for penetration, which required 0.1% sodium azide in the primary solution.

To estimate the number of GABAergic boutons, Stereo Investigator was used to draw contours around the granule cell layer, inner third of the molecular layer, middle third, and outer third. Each layer was analyzed separately. The molecular layer is where dendrites receive 91% of a granule cell's total GABAergic synaptic input (Halasy and Somogyi, 1993). The hilus was not analyzed. At each sample site, a stack of images was collected at 1 μm depth intervals using a 63× Plan-Apochromat 1.4 na lens (Zeiss) and a color camera (1″ CMOS sensor, 3,216 × 2,208). Images were digitally zoomed. Section thickness was measured. Boutons were counted if they appeared in focus for the first time in a 1-μm-thick dissector. This analysis revealed that the estimated number of boutons per dentate gyrus was 1.79 ± 0.88 × 10^9^ (mean ± standard error of mean, range, 1.54–1.94 × 10^9^), which was comparable but less than the reported number of gephyrin-positive punctae (1.99 × 10^9^) and GABA-positive synapses (3.3 × 10^9^) in control rats (Thind et al., 2010). A single GABAergic bouton in the rat dentate gyrus can make multiple synapses (Buckmaster et al., 2016), so the number of boutons would be expected to be less than the number of synapses. The distribution of boutons across layers of the rat dentate gyrus: 0.19 ± 0.06 (mean ± 95% confidence interval of mean) in the granule cell layer, 0.15 ± 0.03 in the inner molecular layer, 0.23 ± 0.02 in the middle molecular layer, and 0.43 ± 0.02 in the outer molecular layer (*p* < 0.001, ANOVA with Holm–Sidak method) was similar to the distribution of gephyrin punctae and GABAergic synapses. The same optical fractionator protocol was used to estimate numbers of GABAergic boutons in sea lions, except the counting grid was larger because sea lion sections were larger. The mean coefficient of error of the stereological analysis was consistently less than the coefficient of variation, indicating sufficient within animal sampling (Table 1; West et al., 1991).

### Statistics

There are four hippocampal groups: controls, bilateral sclerotics, unilateral sclerotics, and unilateral non-sclerotics. Unilateral non-sclerotics are an instructive within-animal control for unilateral sclerotics. Results were analyzed by unpaired two-tailed *t* tests, ANOVA, and Pearson Product Moment Correlation (Systat). Assumptions of normality and equal variance were checked. If verification failed, Mann–Whitney rank sum test or Kruskal–Wallis ANOVA on ranks was used. If differences among groups were greater than would be expected by chance (*p* < 0.05), the Holm–Sidak or Dunn's method was used to isolate the group or groups that differed from the others.

## Results

### Dentate gyrus volume

**Figure 3.**
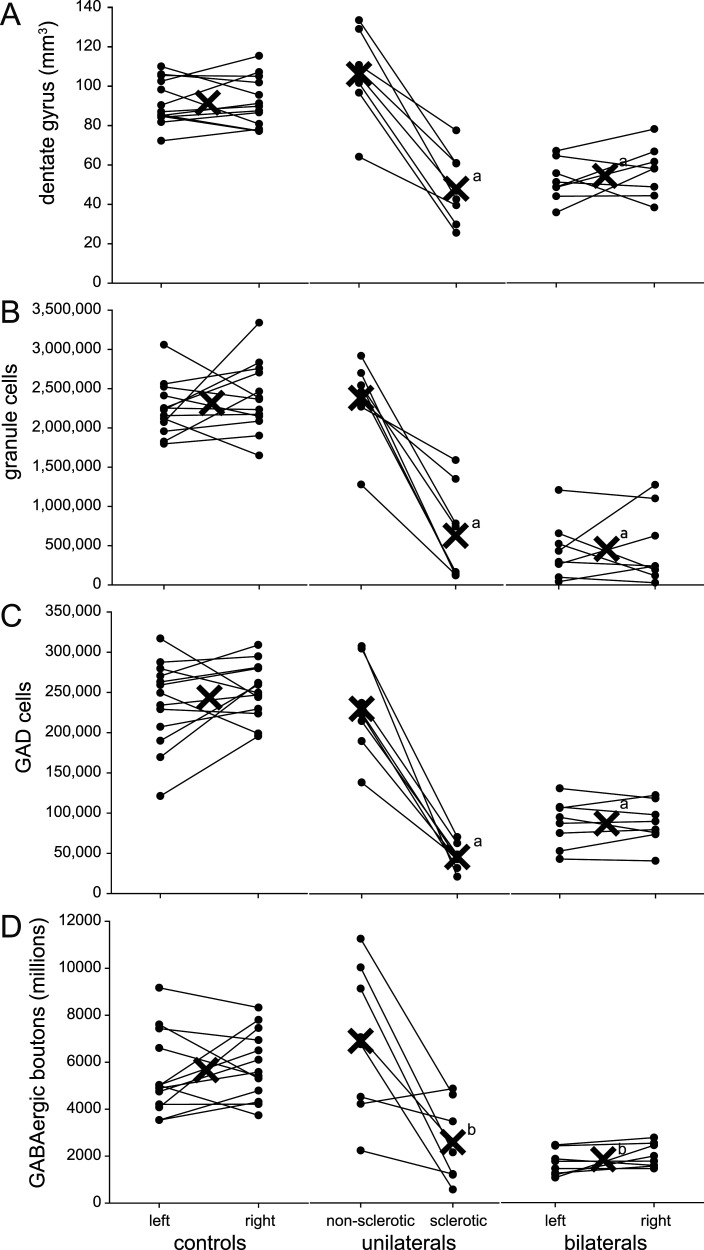
Plots of dentate gyrus volume (***A***), number of granule cells (***B***), glutamic acid decarboxylase (GAD) cells (***C***), and GABAergic boutons per dentate gyrus (***D***) for individual hippocampi of control, unilaterally sclerotic, and bilaterally sclerotic sea lions. Filled circles represent values of individual hippocampi. Lines connect hippocampi from the same sea lion. Xs indicate group averages. Averages for controls and bilateral sclerotic sea lions include both left and right hippocampi. Averages for unilaterally sclerotic sea lions are plotted separately for sclerotic and non-sclerotic hippocampi. ^a^versus control and unilateral non-sclerotic, *p* < 0.001, ANOVA with Holm–Sidak method. ^b^versus control and unilateral non-sclerotic, *p* < 0.05, Kruskal–Wallis ANOVA on ranks with Dunn's method.

The average volume of the dentate gyrus was similarly large in control (92 mm^3^) and unilateral non-sclerotic hippocampi (106 mm^3^; Fig. 3*A*, Table 2). Unilateral sclerotic hippocampi had an average dentate gyrus volume of 48 mm^3^ which was 45% of unilateral non-sclerotic hippocampi (*p* < 0.001, ANOVA). Comparably, bilateral sclerotic hippocampi had an average dentate gyrus volume of 54 mm^3^ which was 59% of control hippocampi (*p* < 0.001). Overall, the average volume of the dentate gyrus in control and unilateral non-sclerotic hippocampi was 97 mm^3^. In sclerotics, the average volume of the dentate gyrus was 51 mm^3^ (47% smaller, *p* < 0.0001, *t* test).

**Table 2.** Dentate gyrus volume and numbers of granule cells, glutamic acid decarboxylase (GAD)-positive interneurons, and γ-amino butyric acid (GABA)ergic boutons per dentate gyrus in control sea lions and sea lions with unilateral or bilateral hippocampal sclerosis

	Control	Unilateral non-sclerotic	Unilateral sclerotic	Bilateral sclerotic
Animals/hippocampi	13/26	8/8	8/8	8/16
Dentate gyrus volume	91.8 ± 6.6	106.1 ± 17.8	47.9^a^ ± 14.6	54.3^a^ ± 7.9
Granule cells	2,318,000 ± 181,000	2,382,000 ± 409,000	627,000^a^ ± 494,000	459,000^a^ ± 309,000
GAD neurons	244,400 ± 23,000	230,100 ± 46,900	45,700^a^ ± 13,100	87,400^a^ ± 22,600
GABAergic boutons (×10^9^)^c^	5.67 ± 0.83	6.90 ± 2.61	2.58^b^ ± 1.34	1.84^b^ ± 0.38

Values represent mean ± 95% confidence interval.

a Versus control and unilateral non-sclerotic, *p* < 0.001, ANOVA with Holm–Sidak method.

b Versus control and unilateral non-sclerotic, *p* < 0.05, Kruskal–Wallis ANOVA on ranks with Dunn's method.

c Includes granule cell layer and molecular layer, not hilus.

### Granule cells

**Figure 4.**
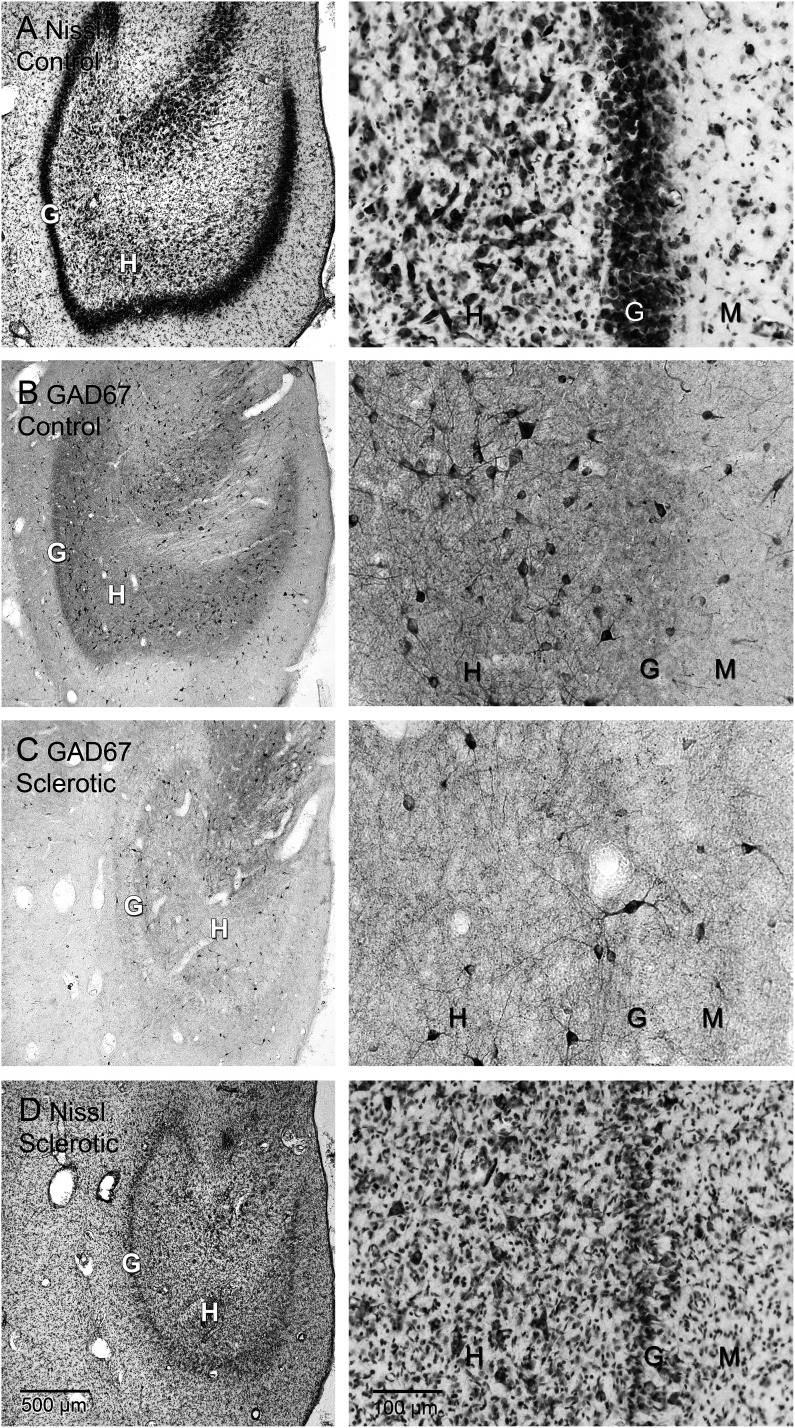
Dentate gyrus of an adult female control (***A***, ***B***) and an adult female bilaterally sclerotic sea lion (***C***, ***D***). Nissl staining (***A***, ***D***) reveals substantial loss of neurons in the hilus (H) and granule cell layer (G) of the sclerotic hippocampus (***D***). ***B***, Glutamic acid decarboxylase (GAD)67-immunoreactivity shows many GAD cells in the hilus of the control sea lion and some in the granule cell layer and molecular layer (M). ***C***, There are fewer GAD cells in the sclerotic hippocampus, especially in the hilus.

As reported previously, the average number of granule cells per dentate gyrus was similar in control (2.32 million) and unilateral non-sclerotic hippocampi (2.38 million; Fig. 3*B*, Table 2). Nissl-stained sections used to estimate granule cell numbers also revealed severe hilar neuron loss that was a defining feature of sclerotic hippocampi (Figs. 4*D*, 6*D*). Unilateral sclerotic hippocampi had an average of 0.63 million granule cells, which was 26% of unilateral non-sclerotic hippocampi (*p* < 0.001, ANOVA). Similarly, bilateral sclerotic hippocampi had an average of 0.46 million granule cells, which was 20% of control hippocampi (*p* < 0.001). Overall, the average number of granule cells in control and unilateral non-sclerotic hippocampi was 2.33 million. In sclerotics it was 0.52 million (78% fewer, *p* < 0.0001, *t* test).

### GAD cells

The average number of GAD cells per dentate gyrus was similar in control (244,400) and unilateral non-sclerotic hippocampi (230,100; Figs. 3*C*, 4; Table 2). Sclerotic hippocampi demonstrated considerable loss of GAD cells. Bilateral sclerotic hippocampi had an average of 87,400 GAD neurons, which was 36% of control hippocampi (*p* < 0.001, ANOVA). Unilateral sclerotic hippocampi had only 45,700 GAD neurons, which was 20% of unilateral non-sclerotic hippocampi (*p* < 0.001). Overall, the average number of GAD cells in control and unilateral non-sclerotic hippocampi was 239,000. In sclerotic hippocampi it was 66,600 (72% loss, *p* < 0.0001, *t* test). These findings reveal substantial loss of GAD cells in the dentate gyrus of sclerotic hippocampi.

**Figure 5.**
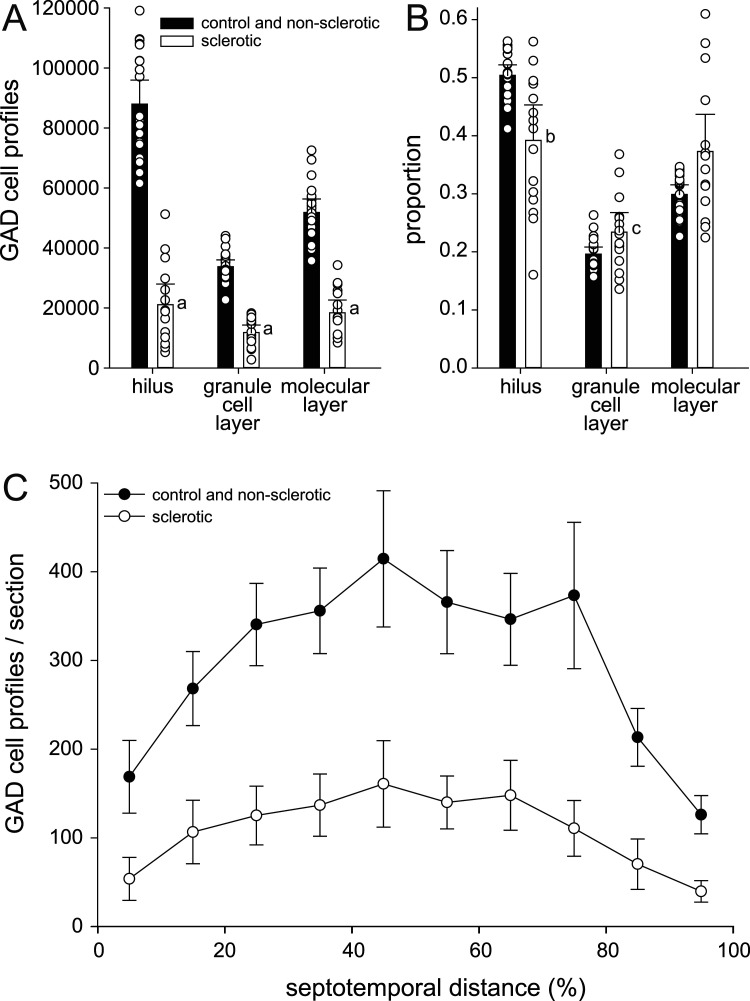
Distribution of glutamic acid decarboxylase (GAD)-immunoreactive cell profiles in layers of the dentate gyrus and along the septotemporal axis of the hippocampus. Number (***A***) and proportion (***B***) of GAD cell profiles in layers of the dentate gyrus. Bars represent averages of non-sclerotic (control and unilateral non-sclerotic) and sclerotic (bilateral and unilateral sclerotic) hippocampi. Markers indicate individuals. Error bars indicate 95% confidence interval. ^a^*p* < 0.0001, *t* test; ^b^*p* = 0.002, ^c^*p* = 0.045, Mann–Whitney rank sum test. ***C***, Number of GAD cell profiles per section along the septotemporal axis in non-sclerotic and sclerotic hippocampi. Values represent mean ± 95% confidence interval. 0% septotemporal distance = septal pole; 100% = temporal pole.

GAD cells were distributed across all layers of the dentate gyrus (Fig. 4). To evaluate GAD cell distribution between dentate gyrus compartments, data from control and unilateral non-sclerotic hippocampi were combined and compared with that of bilateral and unilateral sclerotic hippocampi combined. Left and right hippocampi from control and bilateral hippocampi were averaged yielding a single value per sea lion. In control and non-sclerotic hippocampi, on average 50% of the GAD cell profiles were in the hilus, 20% were in the granule cell layer, and 30% were in the molecular layer (Fig. 5*A*,*B*). In sclerotic hippocampi there were fewer GAD cell profiles in all layers of the dentate gyrus (*p* < 0.0001, *t* tests). Proportionally, compared with non-sclerotics, sclerotic hippocampi had fewer GAD cells in the hilus (*p* = 0.002, Mann–Whitney rank sum test) and more in the granule cell layer (*p* = 0.045). These findings suggest GAD cell loss in sclerotic hippocampi was most severe in the hilus of the dentate gyrus. The proportional loss of GAD cells in sclerotic hippocampi was similar at all septotemporal levels of the hippocampus (Fig. 5*C*).

**Figure 6.**
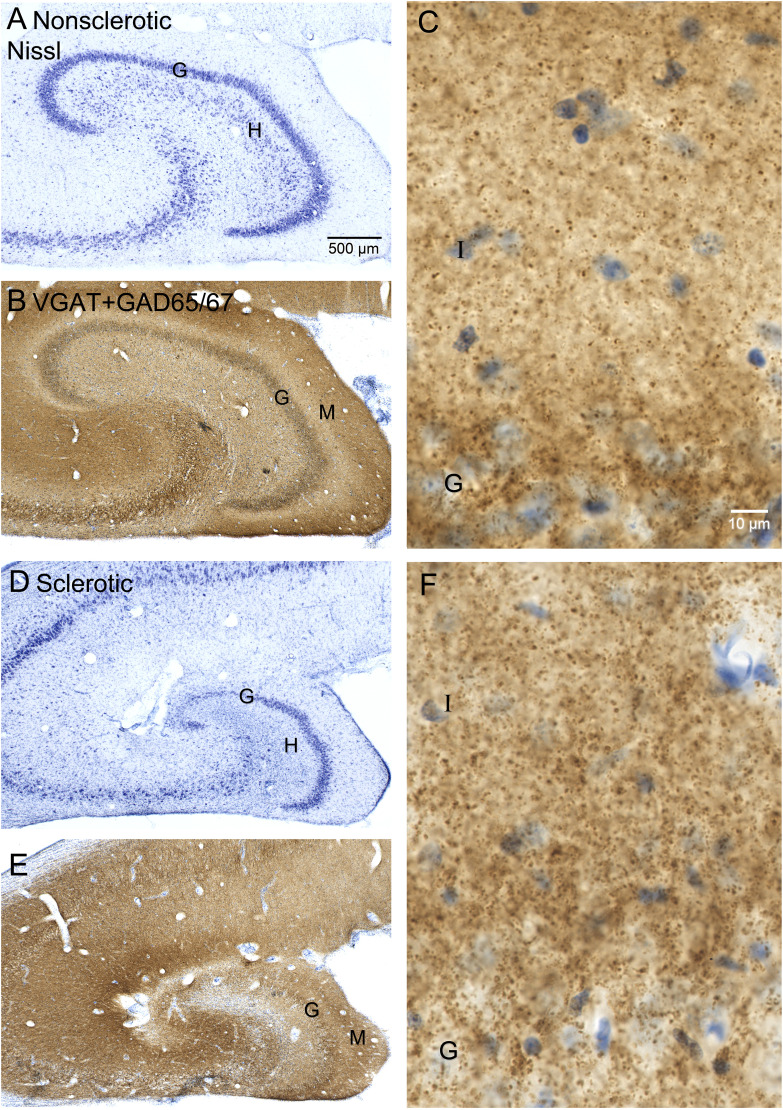
Dentate gyrus of the non-sclerotic (***A–C***) and sclerotic (***D–F***) hippocampi of a subadult female sea lion. Nissl staining (***A***, ***D***) reveals neuron loss in the hilus (H) and granule cell layer (G) of the sclerotic hippocampus. Immunolabeling for γ-amino butyric acid vesicular transporter (VGAT) plus GAD65/67 (***B***, ***C***, ***E***, ***F***) reveals GABAergic synaptic boutons. M, molecular layer; I, inner molecular layer.

### GABAergic boutons

The average number of GABAergic synaptic boutons per dentate gyrus was high in control (5.67 × 10^9^) and unilateral non-sclerotic sea lion hippocampi (6.90 × 10^9^) and low in unilateral sclerotic (2.58 × 10^9^) and bilateral sclerotic hippocampi (1.84 × 10^9^, *p* < 0.001, Kruskal–Wallis ANOVA on ranks; Figs. 3*D*, 6; Table 2). The average number of GABAergic boutons in unilateral sclerotic hippocampi was 37% of unilateral non-sclerotic hippocampi (*p* < 0.05). The average number of GABAergic boutons in bilateral sclerotic hippocampi was 33% of controls (*p* < 0.05). Overall, the average number of boutons in control and unilateral non-sclerotic hippocampi was 5.96 × 10^9^. In sclerotics it was 2.09 × 10^9^ (65% fewer, *p* < 0.0001, *t* test). These findings reveal extensive loss of GABAergic synaptic boutons in sclerotic hippocampi. However, in the sclerotic group the average loss of GABAergic boutons (65% fewer) was not greater than the average loss of granule cells (78% fewer), which is evidence against the hypothesis that bouton loss is disproportionally more severe than granule cell loss in temporal lobe epilepsy.

**Figure 7.**
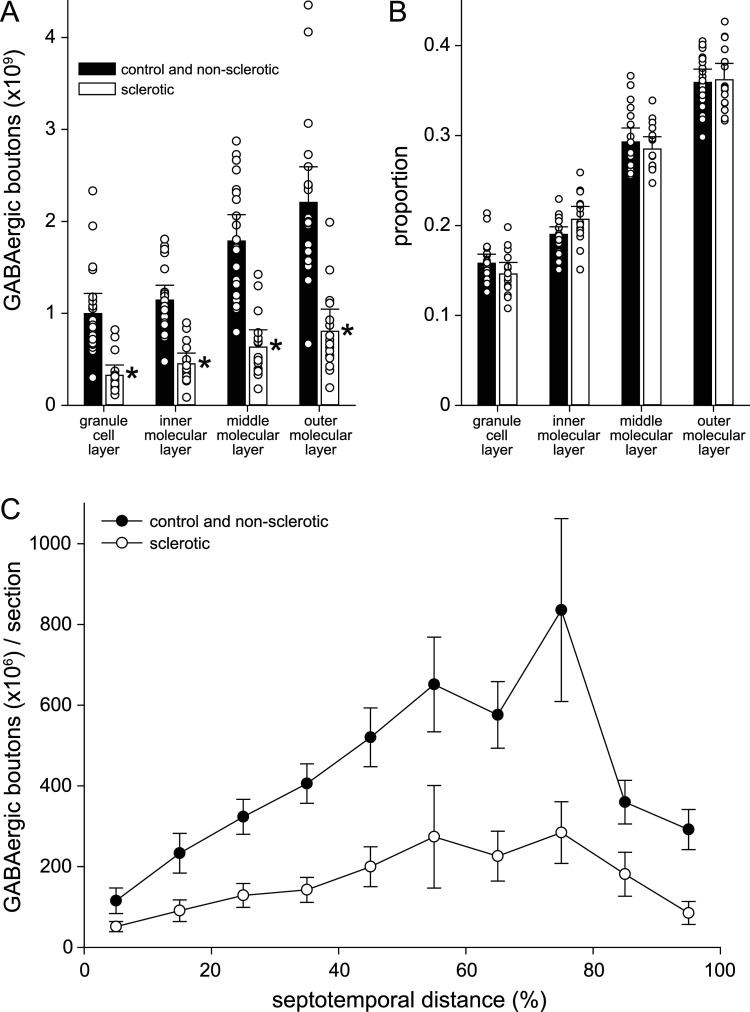
Distribution of γ-amino butyric acid vesicular transporter (VGAT) plus glutamic acid decarboxylase (GAD)65/67-immunoreactive synaptic boutons in layers of the dentate gyrus and along the septotemporal axis of the hippocampus. Number (***A***) and proportion (***B***) of GABAergic boutons in layers of the dentate gyrus. Bars represent averages of non-sclerotic (control and unilateral non-sclerotic) and sclerotic (bilateral and unilateral sclerotic) hippocampi. Markers indicate individuals. Error bars indicate 95% confidence interval. *p < 0.001, Mann–Whitney rank sum test. ***C***, Number of GABAergic boutons per section along the septotemporal axis in non-sclerotic and sclerotic hippocampi. Values represent mean ± 95% confidence interval. 0% septotemporal distance, septal pole; 100%, temporal pole.

In sclerotic hippocampi, loss of GABAergic boutons occurred in the granule cell layer, inner molecular layer, middle molecular layer, and outer molecular layer (Fig. 7*A*). The pattern of bouton distribution across layers of the dentate gyrus was similar in control and non-sclerotic versus sclerotic hippocampi (Fig. 7*B*). Proportionally the fewest boutons were in the granule cell layer (0.14–0.17), then inner molecular layer (0.19–0.21), then middle molecular layer (0.27–0.30), and most were in the outer molecular layer (0.34–0.37; *p* < 0.001, ANOVA with Holm–Sidak method). In sclerotic hippocampi of sea lions, loss of boutons was distributed relatively proportionally along the septotemporal axis of the hippocampus (Fig. 7*C*).

### Correlation testing

Results of the present study revealed smaller volumes of the dentate gyrus, fewer granule cells, fewer GAD cells, and fewer GABAergic boutons in sclerotic hippocampi. To determine if any of these pathological changes were associated with others, correlation testing was performed. In sclerotic hippocampi, there were no significant correlations with the number of GAD cells and dentate gyrus volume (*r* = 0.11, *p* = 0.60, Pearson Moment Correlation), the number of GAD cells and number of granule cells (*r* = −0.19, *p* = 0.38), or the number of GAD cells and the number of GABAergic boutons (*r* = −0.22, *p* = 0.29).

**Figure 8.**
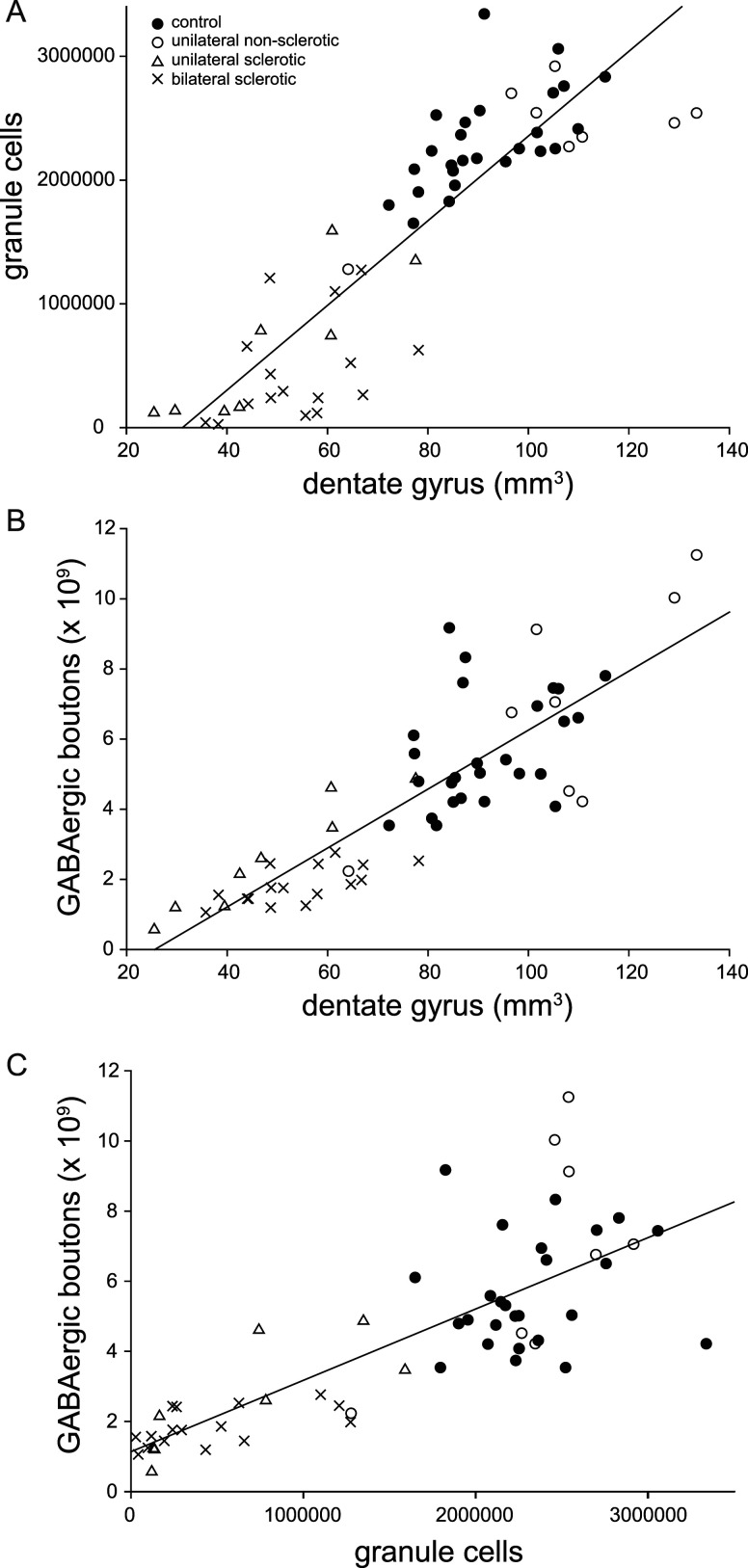
Scatterplots with regression lines of correlated pathological parameters (see text for statistical analysis). Markers indicate values of individual hippocampi. ***A***, Dentate gyrus volume versus number of granule cells. ***B***, Dentate gyrus volume versus number of GABAergic boutons. ***C***, Number of granule cells versus number of GABAergic boutons. The regression line *y*-intercept is 1.14 × 10^9^ boutons, and the slope is 2,030 boutons per granule cell.

There was a significant correlation between dentate gyrus volume and the number of granule cells in sclerotic hippocampi (*r* = 0.56, *p* = 0.005, Pearson Moment Correlation) and in all hippocampi: sclerotic plus non-sclerotic (*r* = 0.88, *p* < 0.0001; Fig. 8*A*). Similarly, there was a significant correlation between dentate gyrus volume and the number of GABAergic boutons in sclerotic (*r* = 0.68, *p* = 0.0003) and all hippocampi (*r* = 0.85, *p* < 0.0001; Fig. 8*B*).

Notably, there was a significant correlation between the number of GABAergic boutons and the number of granule cells in sclerotic (*r* = 0.68, *p* = 0.0002, Pearson Moment Correlation) and all hippocampi (*r* = 0.79, *p* < 0.0001; Fig. 8*C*). This is important as it pertains to the hypothesis being tested. If there had been disproportionate loss of GABAergic boutons in sclerotic hippocampi, as the hypothesis contends, then in the group of sclerotic hippocampi, as the number of granule cells decreased, the number of GABAergic boutons would have declined at a faster rate than in the non-sclerotic group. A regression line through the sclerotic hippocampal group would have had a steeper slope than a regression line for non-sclerotic hippocampi. On the contrary, the slope of a regression line through the sclerotic group (1,530 boutons per granule cell) was slightly less, not greater, than the slope of a regression line through the non-sclerotic group (1,740 boutons per granule cell). These findings do not support the hypothesis of disproportionate loss of GABAergic boutons relative to granule cells in sclerotic hippocampi.

## Discussion

The principal findings of this study are that sea lions with sclerotic hippocampi and temporal lobe epilepsy have a smaller dentate gyrus with fewer granule cells, fewer GAD cells, and fewer GABAergic boutons. Importantly, the number of granule cells and GABAergic boutons are significantly correlated in sclerotic hippocampi, and the loss of boutons is not more severe than the loss of granule cells.

### Motivation

The present study sought to test the hypothesis that in temporal lobe epilepsy there is a disproportionate loss of GABAergic boutons relative to granule cells. Challenges of anatomical research on human patients constrained important and interesting previous human studies (Babb et al., 1989; Wittner et al., 2001; Wittner and Maglóczky, 2017; Alhourani et al., 2020). Sea lions with naturally occurring temporal lobe epilepsy address some limitations of human studies by providing larger sample sizes, superior controls, tissue perfused with fixative immediately after euthanasia, and stereological evaluation of bilateral dentate gyri in their entirety. Another notable improvement is analysis of both somatic and dendritic domains, not just the granule cell layer.

### Dentate gyrus volume and granule cells

The volume of the dentate gyrus in sclerotic hippocampi of epileptic sea lions was approximately half the size of a control. This is similar to the shrinkage of entire hippocampi that was found in sea lions using magnetic resonance imaging (Montie et al., 2010). Dentate gyrus shrinkage correlates with granule cell loss, which is severe in sea lions. In sclerotic hippocampi of human patients, there is an average of 50% loss of granule cells (Babb et al., 1989; Kim et al., 1990; Sass et al., 1990; Mathern et al., 1995, 1996; de Lanerolle et al., 2003; Thom et al., 2005; Blümcke et al., 2007). In contrast, granule cells are generally preserved in epileptic rodents (Thind et al., 2010; Buckmaster and Lew, 2011), and while there are some reports of reduced granule cell density in chronic rodent models (Mello et al., 1993; Cavazos et al., 1994; Mathern et al., 1997; Mathern and Bertram, 2021), they do not approach the reductions in sea lions or humans.

### GAD cells

GAD cell loss was substantial in epileptic sea lions with sclerotic hippocampi. Loss of GAD cells raises the possibility that granule cells lose inhibitory synaptic input, become hyperexcitable, and cause seizures. Like epileptic sea lions, rat models of temporal lobe epilepsy display GAD cell loss in the dentate gyrus (Obenaus et al., 1993; Houser and Esclapez, 1996; Buckmaster and Jongen-Rêlo, 1999). In contrast, preservation of GAD cells in the dentate gyrus of patients with temporal lobe epilepsy has been reported (Babb et al., 1989; Mathern et al., 1995). The difference might be attributable to cell counting methods (density measures vs stereology, given granule cell dispersion), sensitivity of GAD antibodies, tissue processing protocols, and/or quality or type of control tissue. Previous studies of the dentate gyrus in sclerotic hippocampi in patients with temporal lobe epilepsy used antibodies for markers of various cell types and found interneuron loss (Sloviter et al., 1991; Zhu et al., 1997; Maglóczky et al., 2000; Andrioli et al., 2007; Tóth et al., 2010), including somatostatin cells (de Lanerolle et al., 1989; Mathern et al., 1995). In sea lions, somatostatin cell loss (Buckmaster et al., 2014) could be an important contributor to GAD cell loss, which was most severe in the hilus.

### GABAergic boutons

GABAergic bouton numbers were severely reduced in the dentate gyrus of sea lions with temporal lobe epilepsy. In a number of different rodent models of chronic temporal lobe epilepsy following an initial decrease in the first week after status epilepticus, there is an increase in the labeling of GABAergic boutons in the inner, outer, or entire molecular layer (Davenport et al., 1990; Houser and Esclapez, 1996; Mathern et al., 1997; Esclapez and Houser, 1999; André et al., 2001), which was stereologically quantified by Thind et al. (2010). In dentate gyrus resected from humans with temporal lobe epilepsy, the number of GABAergic boutons and synapses per granule cell, or their density, are increased in the granule cell layer (Babb et al., 1989; Wittner et al., 2001; Wittner and Maglóczky, 2017; Alhourani et al., 2020). These findings from patients and rodents suggest that GABAergic synapses with granule cells are increased, not reduced, in temporal lobe epilepsy. Given that the loss of granule cells was more severe than the loss of GABAergic boutons in epileptic sea lions, results of the present study are consistent with more GABAergic boutons per granule cell in epileptic patients and rodent models.

Numbers of GABAergic boutons and granule cells were correlated in sclerotic hippocampi of sea lions. It is unclear whether the correlation was present immediately after the precipitating injury or if axon sprouting resulted in the correlation. If there were GABAergic axon sprouting, it could come from local (Zhang et al., 2009; Peng et al., 2013) or extrahippocampal (Soussi et al., 2015) GAD cells. While GABAergic bouton loss was correlated with granule cell loss, it was not correlated with GAD cell loss. These findings suggest that following a precipitating injury, if GABAergic axon sprouting occurs, then it might be influenced more by the availability of granule cell synaptic targets than by the number of GAD cells in the dentate gyrus.

### Limitations

Limitations of the present study include those of the sea lion model that have been discussed (Buckmaster, 2017). Immunolabeling GAD cells can be challenging (Obenaus et al., 1993; Houser and Esclapez, 1996). Attempts to use in situ hybridization on sea lion tissue were unsuccessful, but after protocols were optimized, GAD immunostaining revealed well-labeled cells at numbers proportionally appropriate compared with rats processed with in situ hybridization for GAD (Buckmaster and Jongen-Rêlo, 1999; Thind et al., 2010). Quantifying synaptic boutons was challenging. Boutons are small, densely packed, and sometimes overlapping in sections. A new camera and lens were used to better visualize boutons, and a new optical fractionator protocol was developed that generated stacked and zoomed images. The new methods yielded results from rat tissue consistent with a previous rat study that used confocal and electron microscopic approaches to label and quantify GABAergic synapses (Thind et al., 2010). Results from control sea lion tissue were proportionally appropriate compared with bouton results of rats in the present study. Sea lion GABAergic synaptic bouton counts in the present study suggest previous analyses of parvalbumin- (Cameron et al., 2019) and cannabinoid receptor 1-labeled boutons in sea lions (Seelman et al., 2022) that were analyzed with other methods might have been underestimates. However, the relative comparisons of control and epileptic groups should be valid as all groups were evaluated with identical techniques in those studies.

While the present study quantified GABAergic bouton numbers in the granule cell and molecular layers of the dentate gyrus, how many synapses each bouton made and which cell types they targeted were not certain. Adding to the uncertainty, reorganization of synaptic input to granule cells (Du et al., 2017) and interneurons (Sloviter et al., 2012) is reported in rodent models of temporal lobe epilepsy. Besides granule cells, potential synaptic targets include interneurons in the molecular and granule cell layers and dendrites from some hilar interneurons and mossy cells that extend into the molecular layer. However, many hilar GAD cell and mossy cell dendrites remain confined to the hilus and do not extend into the granule cell and molecular layers. Furthermore, hilar neuron numbers are substantially reduced in sea lions with sclerotic hippocampi (Buckmaster et al., 2014). For a conservative quantitative estimate, potential nongranule cell targets can be overestimated by assuming that all surviving hilar neurons (quantified in the 2014 study) and interneurons were synaptic targets. Even then, sclerotic hippocampi would be estimated to have over five times more granule cells than all other potential target neurons combined. Thus, granule cells remained the most abundant synaptic target of GABAergic boutons in the granule cell and molecular layers of sclerotic hippocampi.

### Conclusion

Data of the present study show that numbers of granule cells and GABAergic boutons were reduced and correlated in sclerotic hippocampi. Other sea lion studies that used interneuron subtype specific markers find no disproportionate loss of GABAergic boutons (Cameron et al., 2019; Seelman et al., 2022). Similarly, studies of human patients with temporal lobe epilepsy and rodent models report no disproportionate loss of GABAergic boutons or inhibitory synapses (Babb et al., 1989; Wittner et al., 2001; Thind et al., 2010; Wittner and Maglóczky, 2017; Alhourani et al., 2020). On the contrary, they report more boutons per granule cell. Together, these findings contradict the hypothesis that disproportionate reductions in GABAergic boutons and synapses reduce inhibition of granule cells. What then reduces inhibition of granule cells in patients and animal models of temporal lobe epilepsy (see Introduction)? If in epileptic hippocampi GABAergic boutons are in place at sufficient numbers but dysfunctional (Zhang and Buckmaster, 2009), it might be possible to reestablish synaptic function and provide seizure relief.

## Synthesis

Reviewing Editor: Viji Santhakumar, University of California Riverside Decisions are customarily a result of the Reviewing Editor and the peer reviewers coming together and discussing their recommendations until a consensus is reached. When revisions are invited, a fact-based synthesis statement explaining their decision and outlining what is needed to prepare a revision will be listed below. The following reviewer(s) agreed to reveal their identity: Suchitra Joshi.

The manuscript has addressed most major concerns. Some minor edits and clarifications will help improve the manuscript.

In the abstract, we recommend you remove the last part of the last sentence "suggesting preservation of a critical anatomical inhibitory relationship in sclerotic hippocampi." Indeed, if the loss of GABAergic boutons follows the loss of dentate granule cells, it is far too much to suggest "preservation of a critical anatomical relationship". As known by the authors, there is a huge reorganization of the dentate neuronal network, including cell loss of GABAergic neurons in the hilus of the dentate gyrus as described in this study, sprouting of mossy fibers, and modification of membrane receptors that strongly suggest that the anatomical connectivity is deeply reshaped and not preserved. See also the comment for the discussion.

In the results (Lines 270-271): It is mentioned that in "In control rats, the proportional distribution of GABAergic boutons was similar except more boutons were in the granule cell layer (0.19) and outer molecular layer (0.43)". Are these differences significant? If these are mentioned, they should be discussed.

Similarly, results (Lines 279-282) indicate that "there were no significant correlations between ........the number of GAD cells and the number of GABAergic boutons (r=−0.22, p=0.29). This interesting data is not fully discussed. It could suggest sprouting of surviving GABAergic neurons, as mentioned by the authors. It may also indicate sprouting from long-distance GABAergic innervation reported also in the literature in rodent models of TLE, such as from the septum or the hypothalamic supramammillary nucleus, which display a dual GABAergic and glutamatergic phenotype. The remodeling of this GABAergic innervation could clearly modify the inhibitory input on dentate granule cells.

Please consider moving Lines 292- 302 in the discussion section to the results section. "This is important because it pertains to the hypothesis being tested. If there had been disproportionate loss of GABAergic boutons in sclerotic hippocampi, as the hypothesis contends, then in the group of sclerotic hippocampi, as the number of granule cells decreased, the number of GABAergic boutons would have declined at a faster rate than in the non-sclerotic group. That would have been evidence of disproportionate loss of GABAergic boutons. A regression line through the sclerotic hippocampal group would have had a steeper slope than a regression line for non-sclerotic hippocampi. On the contrary, the slope of a regression line through the non-sclerotic group (1530 boutons per granule cell) was slightly less, not greater, than the slope of a regression line through the non-sclerotic group (1740 boutons per granule cell). These findings do not support the hypothesis of disproportionate loss of GABAergic boutons relative to granule cells in sclerotic hippocampi".

The discussion of data obtained from studies performed in rodent models or hippocampal tissue from TLE patients are far too long. Please briefly mention the similarities or differences between their results and the data reported in rodent models or in hippocampal tissue from TLE patients.

Similarly, discussion of the limitations of working with rodent models and brain tissue from TLE patients is far too long and can be made more concise.

Please discuss findings regarding the lack of correlation between the loss of GABAergic cells in the DG and the loss of GABAergic boutons.

Consider removing the following sentences

Lines: 373-376: "However, rodents do not perfectly model human temporal lobe epilepsy. Status epilepticus after systemic treatment with pilocarpine, like kainate (Buckmaster and Dudek, 1997), consistently causes bilateral hippocampal neuron loss in rodents. Human patients, on the other hand, frequently have unilateral disease (Briellman et al., 1999)".

Lines 379-382: "In pilocarpine-treated rats, granule cell numbers decrease to 80% of controls (20% loss) at after status epilepticus but recover to normal levels in chronically epileptic animals (Thind et al., 2010). Thus, rodent models do not replicate the pathology of persistent granule cell loss seen in patients". It is to be noted that rodent models are not limited to the pilocarpine model and/or to data from a single study.
